# Unraveling Stage-Dependent Expression Patterns of Circular RNAs and Their Related ceRNA Modulation in Ovine Postnatal Testis Development

**DOI:** 10.3389/fcell.2021.627439

**Published:** 2021-03-19

**Authors:** Taotao Li, Ruirui Luo, Xia Wang, Huihui Wang, Xingxu Zhao, Yunxia Guo, Haitao Jiang, Youji Ma

**Affiliations:** ^1^College of Animal Science and Technology, Gansu Agricultural University, Lanzhou, China; ^2^College of Veterinary Medicine, Gansu Agricultural University, Lanzhou, China; ^3^College of Life Science, Hebei Agricultural University, Baoding, China; ^4^Wenshang County Inspection and Testing Center, Jining, China

**Keywords:** circRNAs, sheep, testis, spermatogenesis, ceRNA

## Abstract

Circular RNAs (circRNAs) have been shown to function in the reproductive systems including testis. However, their expression, as well as function in testicular development of sheep remain undefined. Herein, we performed RNA sequencing to reveal circRNA temporal expression patterns in testes of Tibetan sheep from different stages of maturation (3M, 3-month-old; 1Y, 1-year-old; 3Y, 3-year-old). A total of 3,982, 414, and 4,060 differentially expressed (DE) circRNAs were uncovered from 3M vs 1Y, 1Y vs 3Y, and 3M vs 3Y, respectively. Functional enrichment assessment indicated that the source genes of DE circRNAs were primarily engaged in spermatogenesis and testicular immune privilege including blood–testis barrier (BTB). We subsequently constructed the core circRNA–miRNA–mRNA interaction network for genes related to testicular function, such as spermatogenesis, germ cell development, BTB, and cell cycle/meiosis. Furthermore, we validated the target associations between either circ_024949, circ_026259 or IGF1, and oar-miR-29b in this network, and revealed their similar expression signatures in developmental testes that they were extensively expressed in germ cells, Leydig cells, and Sertoli cells, thus suggesting their broad functions in the functional maintenance of Leydig cells and Sertoli cells, as well as the development and maturation of male germ cells. Meanwhile, circ_026259 was shown to promote IGF1 expression through inhibition of oar-miR-29b in sheep Sertoli cells. This work gives the first global view for the expression and regulation of circRNAs in sheep testis, which will be helpful for providing new insights into the molecular mechanism of ovine testis function.

## Introduction

The testis is the only specific organ known to be capable of generating male gametes (spermatozoa), essential for male fertility. In mammals, spermatozoa present in the testis are generated by spermatogenesis, which is subdivided into three distinct phases: mitotic renewal, proliferation, and differentiation of spermatogonia, meiotic progression of spermatocytes, as well as differentiation of haploid spermatids (Zhou R. et al., 2019). Spermatogenesis is a highly coordinated, precise, and dynamic cell differentiation process that is reflected in the complex testicular gene modulation and gene expression processes. The genome of testicular cells is actively transcribed into RNAs that besides protein-coding messenger RNAs (mRNAs), involves many non-coding RNAs consisting of circular RNAs (circRNAs), as well as microRNAs (miRNAs) to regulate and yield the phase-specific gene expression patterns (Kotaja, 2014; Hu et al., 2018).

Circular RNAs are a prominent class of newly discovered closed circular RNA molecules in eukaryotes generated by the alternative shearing of precursor mRNAs (Li et al., 2018). One of the major functions of circRNAs is to modulate miRNA activity by functioning as either a competitive endogenous RNA (ceRNA) or as an miRNA sponge, consequently modulating the gene expression (Zhong et al., 2018). Existing literature shows that circRNAs are participated in diverse biological processes in mammals such as growth (Zhang P. et al., 2019), development (Salzman, 2016; Zhang P. et al., 2019), reproduction (Zhou F. et al., 2019), and immunity (Yang et al., 2018). For instance, circRNA-9119 can function as a endogenous sponge or ceRNA to upregulate the expression of RIG-I and TLR3 in mouse testicular somatic cells (Sertoli cells and Leydig cells) through competitive binding with miR-26a/miR-136, thus contributing to the pro-inflammatory activities in orchitis (Qin et al., 2019). In sheep, mounting research evidence has also shown circRNAs are abundant in the transcriptome of ovine tissues as well like mammary gland (Hao et al., 2020), pituitary (Li et al., 2019), uterus (La et al., 2020), and muscle (Cao et al., 2018), but it is not known whether this exists and functions in ovine testis.

Tibetan sheep (*Ovis aries*), a well-known Chinese domestic sheep breed, is mainly distributed in Qinghai-Tibet Plateau (QTP) over 3,000 m above sea level, which provides food and livelihoods to the local farmers and herdsmen (Wang et al., 2019), and serves a pivotal role in the QTP ecosystem functions (Jing et al., 2020). Due to the long-term excessive dependence on grazing that lacks supplementary feeding, the main reproductive characteristics of Tibetan sheep are late sexual maturity and low reproductive rates. Hence, insight into the underlying mechanisms of testicular development of male Tibetan sheep is of great significance for the reproductive biology of sheep. Here, we assume that the expressions and functions of these genes are partially or entirely regulated by circRNAs. In order to validate this hypothesis, herein, we explored the dynamic circRNA expression profiling in developmental Tibetan sheep testes at distinct reproductive stages with the aid of high-throughput RNA sequencing (RNA-seq) technology.

## Materials and Methods

### Animals

Overall, 24 healthy male Tibetan sheep (Ganjia, Xiahe, China; altitude >3,000 m) were used in study, divided in three age groups (*n* = 8 for each age group): 3-month-old (3 M; sexually immature), 1-year-old (1Y; sexually mature), and 3-year-old (3Y; adult). The right testes were harvested immediately after sacrifice of all animals, briefly washed with PBS, and deep-frozen quickly in liquid nitrogen for RNA and protein extraction or fixed in 4% paraformaldehyde for making paraffin sections.

### RNA Isolation, Library Preparation, and circRNA Sequencing

The Trizol reagent (Invitrogen, United States) was employed to extract the total RNA and their concentration and quality were explored by NanoDrop Spectrophotometer (NanoDrop, United States), Agilent 2100 Bioanalyzer (Agilent Technologies, United States), as well as RNase free agarose gel electrophoresis. Of eight RNA samples for each age group, four were selected randomly for RNA-seq, and all samples were used for qPCR validations. For RNA-seq, ribosomal RNA (rRNA) was depleted with a Ribo-Zero Gold rRNA Removal Kit (Illumina, United States). The remaining RNAs were used to construct the strand specific sequencing library. Briefly, RNAs were fragmented (approximately 200 bp), then converted into 1st-strand cDNA via reverse transcription with random hexamer primers. Next, the 2nd-strand cDNA was processed with dNTPs, RNase H, DNA polymerase I, and buffer. QiaQuick PCR was used to purify the cDNA fragments, and then end repaired and base A introduced, and ligated into Illumina sequencing adapters. Then, the 2nd-strand cDNA was depleted by the UNG enzyme (Uracil-N-Glycosylase). The digested products were size-selected and PCR amplified to generate RNA-seq libraries, followed by sequencing on an Illumina HiSeq4000 system (150-bp paired-end reads; Illumina, United States) at Gene *Denovo* Co., Guangzhou, China.

### Identification of circRNAs

The clean reads were acquired by discarding low-quality reads, including reads with adapters, reads bearing greater than 10% of unknown bases (N) and reads with greater than 50% of low-quality (Q-value ≤ 20) bases. We mapped the resulting high-quality reads to the sheep Oar_v4.0 reference genome using TopHat2 (Kim et al., 2013). The 20 bp sequences from the two ends for each unmapped read (Anchors reads) were re-aligned to the reference genome with bowtie2 aligner, and subsequently submitted to Find_Circ software (Memczak et al., 2013) with default parameters to identify circRNA.

### Functional Annotation and Enrichment Analysis of circRNA Source Genes

The previous literature review showed that one of the functions of CircRNAs is realized through modulating the expression of its source gene (Qu et al., 2015; Shao et al., 2021). To interpret the potential biological roles of circRNAs, Gene Ontology (GO) annotation, as well as Kyoto Encyclopedia of Genes and Genomes (KEGG) pathway analysis of circRNA source genes were thus carried out using GO web resource (http://www.geneontology.org/) and KEGG web portal (http://www.genome.jp/kegg/pathway.html).

### Quantification of circRNA Abundance and Differential Expression Evaluation

The expression abundance of the identified circRNAs was computed by RPM (reads per million mapping) method. EdgeR package in R^1^ was employed to obtain the differentially expressed (DE) circRNAs between every two age groups. The circRNAs with a | fold change| > 2.0 and *p*-value <0.05 were regarded as significant differentially expressed.

### Integrated Analysis of circRNAs–miRNAs–mRNAs

Based on our previous Illumina HiSeq miRNA and mRNA sequencing data from the same samples (NCBI SRA accessions for mRNA sequencing data: SRR11397689-SRR11397700; NCBI SRA accessions for mRNA sequencing data: SRR11348536-SRR11348547), the associations between circRNAs/mRNAs and miRNAs were estimated by using Mireap,^2^ TargetScan 7.0,^3^ as well as Miranda.^4^ CircRNA-miRNA-mRNA ceRNA regulatory network was built as per the following criteria: (1) Expression association between circRNA-miRNA or mRNA-miRNA was assessed by Spearman rank correlation coefficient (SCC); we selected the pairs with SCC < −0.7 as negatively co-expressed circRNA–miRNA pairs or mRNA–miRNA pairs. (2) Expression association between circRNA-mRNA was determined via Pearson correlation coefficient (PCC); we selected the pairs with PCC > 0.9 as co-expressed circRNA–mRNA pairs. (3) The *p*-value was computed based on a hypergeometric cumulative distribution function test to assess shared miRNA sponges between circRNA–mRNA; we chose the pairs with a *p*-value of <0.05 as final ceRNA pairs. For these mRNAs in network, GO and KEGG enrichment assessment were conducted to further elucidate the function of circRNAs. The ceRNA network related to testicular function was visualized by Cytoscape 3.8.

### Dual Luciferase Reporter

The wild-type (WT) and mutant-type (MUT) circ_024949, circ_026259, and IGF1 3′-UTR containing the putative binding site of oar-miR-29b were purchased from GENEWIZ (Suzhou, China) and subsequently inserted into the pmirGLO luciferase reporter vector *Xho*I/*Sal*I sites (Promega, United States). Oar-miR-29b mimic and mimic negative control (NC) were bought by Jima (Shanghai, China), and their sequences are provided in Supplementary Table 1. The luciferase reporter plasmids (circ_024949 WT, circ_024949 MUT, circ_026259 WT, circ_026259 MUT, IGF1 3′-UTR WT, or IGF1 3′-UTR MUT) were transfected with oar-miR-29b mimic or mimic NC into 293T cells using Lipofectamine 2000 transfection vehicle (Invitrogen, Carlsbad, CA, United States) as per the instructions provided by vendors. The firefly, as well as renilla luciferase activities were examined at 48 h after transfection by the Dual-Luciferase Reporter Assay System (Promega, United States). The relative luciferase enzyme activity was shown as ratios of firefly luciferase to renilla luciferase. Three repeated experiments were set up in each group.

### Isolation, Culturing, and Transfection of Sheep Sertoli Cells

Testes were collected from healthy Tibetan sheep aged three-month-old, sterilized with 75% alcohol and then transported to the laboratory within 30 min in PBS buffer containing penicillin and streptomycin on ice. Sertoli cells were isolated as described recently (Gao et al., 2020), with some alterations. Briefly, under aseptic conditions, testes were rinsed twice with PBS after removal of the tunica albuginea, and seminiferous tubules were minced into paste and digested with type IV collagenase (1 mg/mL) for 1 h at 37°C. After centrifugation, the supernatant was discarded and digested with 0.25% trypsin for 20 min at 37°C. The digestion was terminated with DMEM/F12 supplemented with 10% FBS (Gibco, United States), and the digested cells were filtered through 100-, 200-, and 300-mesh screen. After centrifugation, cells were washed with PBS, resuspended in high-glucose DMEM containing 10% FBS, seeded in culture flasks, and routinely cultured at 37°C in 5 % CO_2_. After 6 h of culture, the medium was removed and changed with fresh medium to discard the suspending germ cells. After a continued culture time of 18 h, the supernatant was discarded and 0.05% trypsin was added to transiently digest cells before addition of fresh medium. When the cells reached ∼80% confluency, the above steps were repeated 3–4 times to remove residual germ cells and to obtain pure Sertoli cells. The isolated sheep Sertoli cells were identified by RT-PCR and immunofluorescence staining with antibodies against Vimentin and SOX9.

The linear sequence of circ_026259 derived from KLHL5 gene was synthesized and cloned into the *Eco*RI and *Bam*HI restriction sites of overexpression vector pCD25-ciR (pCD25-circ_026259; Geneseed, Guangzhou, China), which included a forward and backward circular frame to promote circularization. Small interfering RNAs (siRNAs) targeting circ_026259 (si1-circ_026259 and si2-circ_026259), oar-miR-29b inhibitor, or the corresponding negative controls (si-NC and inhibitor NC) were provided by Jima (Shanghai, China), and their sequences are listed in Supplementary Table 1. For transient transfections, Sertoli cells were seeded in culture plates in a proper density and 24 h later transfected with pCD25-circ_026259, control vector (pCD25-circ), si1-circ_026259, si2-circ_026259, si-NC, oar-miR-29b mimic/inhibitor, or mimic/inhibitor NC using Lipofectamine 2000 reagent (Invitrogen) per as recommended by the provider. Cells were harvested 48 h after transfection for assessment of the transfection efficiency and subsequent analysis.

### PCR Analysis

The total RNA was isolated from cells and tissues with TRIzol reagent (TransGen, Beijing, China), and reverse transcribed to cDNA using an Evo M-MLV RT Kit with gDNA Clean for qPCR (Accurate, Hunan, China). The cytoplasmic and nuclear RNAs from Sertoli cells were prepared using PARIS^TM^ kit (Invitrogen) as per the instructions provided by vendors. For RNase R treatment, 1 μg total RNA was incubated for 30 min at 37°C with 1μL RNase R (20 U; Epicentre, United States), and then deactivated RNase R at 80°C for 10 min. RT-PCR was carried out using 2× Pro Taq Master Mix kit (Accurate) as the per manufacturer’s instructions. qPCR was done under the following conditions: 95°C for 30 s, then 40 two-step amplification cycles of 95°C for 5 s and 60°C for 30 s. Divergent primers used for amplification of circRNA-specific back splice junctions were designed. All primers used were synthesized by Tsingke (Xi’an, China). Details of the primers used are presented in Supplementary Table 2. β-actin or GAPDH was used as an internal reference gene for circRNA and mRNA, and U6, for miRNA. The expression levels relative to internal reference gene were computed by 2^–ΔΔCt^ approach. PCR products were confirmed via electrophoresis on 1.5% agarose gels was employed to confirm the PCR products and bands were extracted and subjected to Sanger sequencing.

### *In situ* Hybridization and Immunofluorescence

Digoxigenin (DIG)-labeled RNA antisense probes for circ_024949 (5′-ATATTGATAGAGAGCTTT CCCTAAATGTGT-3′), circ_026259 (5′-TACTATTGGAAG TCCTGTGGTGCAAGAAG-3′), oar-miR-29b (5′-ACACTGA TTTCAAATGGTGCTA-3′), and IGF1 (5′-TGAA ATAAAAGCCCCTGTCTCCGCACACGAA-3′) were purchased from General Biosystems (Anhui, China) for *in situ* hybridization. After conventional dewaxing and rehydration, 5-μm paraffin sections or fixed Sertoli cells were digested in proteinase K solution and bloked with 3% H_2_O_2_/methanol. Pre-hybridization, hybridization and washing were conducted according to standard methods. After this step, probe signals were visualized with mouse anti-DIG antibody (HRP-conjugated) and direct FITC-TSA kit (Servicebio, Wuhan, China). The nuclei were counterstained by DAPI. Cells or the adjacent sections were hybridized with sense probes and without probes to produce negative controls. For immunofluorescence staining of testis tissue sections, sections were blocked with 5% BSA (Solarbio, Beijing, China) prior to incubation with rabbit anti-IGF1 polyclonal antibody (1:200; Bioss, Beijing, China) overnight at 4°C. Then, sections were added with goat anti-rabbit IgG labeled with CY3 (1:200; Servicebio, Wuhan, China) and counterstained with DAPI. The sections were seen in Nikon fluorescence microscope (Eclipse C1, Tokyo, Japan) and imaged with CaseViewer software. For immunofluorescence cell staining, Sertoli cells were fixed, permeabilized, and subsequently incubated with primary antibodies against Vimentin (1:200; Bioss, China) or SOX9 (1:200; Bioss, China) followed by incubation with FITC-conjugated secondary antibody. The remaining procedures were the same as above.

### Western Blot

Proteins from testes and Sertoli cells were extracted with RIPA buffer and separated on 12% SDS-PAGE gel. After transfer, the PVDF blot membranes were blocked and then probed with rabbit polyclonal antibody against IGF1 (1: 500, Bioss, Beijing, China) at 4°C overnight. Anti-β-actin polyclonal antibody (1:1,500, Bioss, Beijing, China) was used as an internal reference. These blots were further conjugated with a goat anti-rabbit IgG secondary antibody labeled with HRP (1: 5,000, Bioss, Beijing, China) via incubation and revealed with ECL kit (NCM Biotech, Suzhou, China) and exposed to X-ray films. Quantification of the blot intensities was performed using the AlphaEaseFC software.

### Statistics

Data among/between groups were analyzed using SPSS 21.0 software (SPSS, Inc., Chicago, IL, United States) with one-way ANOVA followed by LSD *post hoc* test, or the independent samples t test; the results are indicated as the mean ± SD. The differences between two groups were marked by ^∗^*P* < 0.05 and ^∗∗^*P* < 0.01.

## Results

### Overview of circRNA Sequencing

**FIGURE 1:**
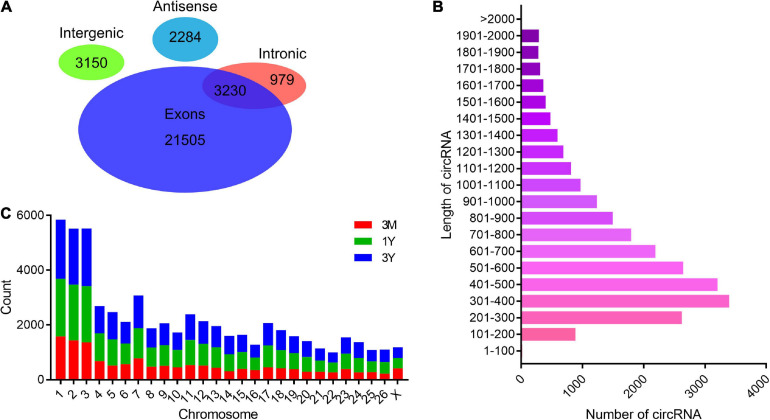
General features of circRNAs. **(A)** Venn diagram indicating that circRNAs numbers originated from different genomic sites. **(B)** Length distributions for the identified circRNAs. **(C)** Chromosome distribution map of circRNAs. 3M, 1Y, and 3Y refer respectively to 3-month-old, 1-year-old, and 3-year-old.

After the raw reads were filtered, a total of 994,655,502 high-quality clean reads (85,301,018 from 3M group, 78,331,884 from 1Y group, and 85,030,974 from 3Y group) were obtained. There were 14.88, 17.46, and 18.67% of the high-quality reads that were not aligned to the sheep reference genome in 3M, 1Y, and 3Y groups, respectively, and 20 bp sequences (Anchors reads) on both ends of which were selected for re-alignment with the reference genome to identify circRNA. Specific information regarding the overall analysis of the sequencing data is presented in Supplementary Table 3. RNA-seq data has been deposited to the SRA database in NCBI (SRA accessions: SRR11348536-SRR11348547). In total, 31,148 circRNAs were identified in this study, which were named and numbered from circ_000001 to circ_031148. According to their position on the ovine genome, the vast majority of these circRNAs were showed to have originated from protein-coding exons of genes, while many others were originated from antisense or intronic sites (Figure 1A). Most of the circRNAs were between 200 and 700 nt long, with 400 nt being the most abundant length (Figure 1B). These circRNAs were distributed on all chromosomes including 26 autosomes and X chromosome, but were mainly existed in chromosomes 1, 2, and 3 (Figure 1C).

### Differential Expression Analysis

**FIGURE 2:**
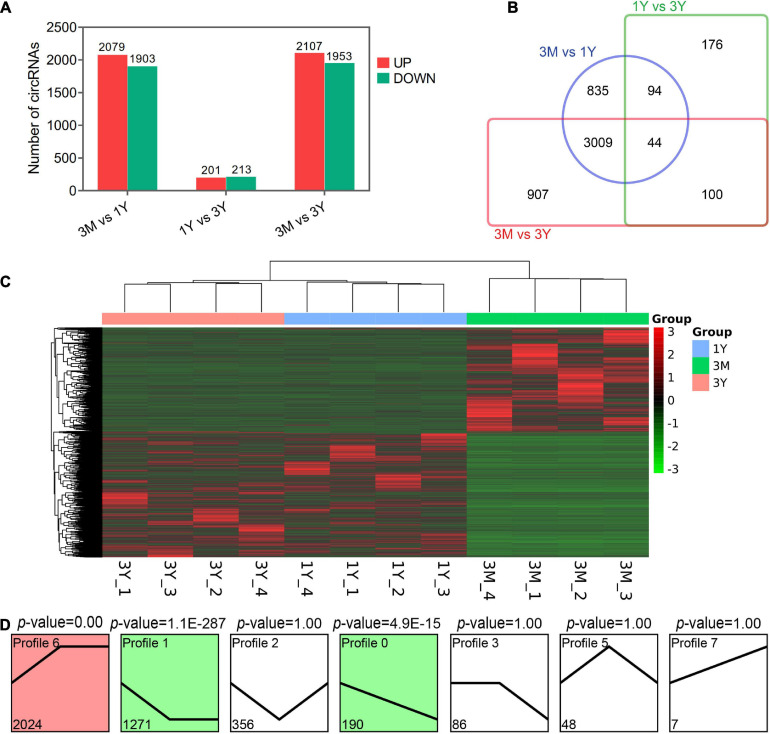
Summary of differential expression analysis of the annotated circRNAs. **(A)** Statistics of differential circRNAs. **(B)** A Venn diagram indicating the shared and unique differential circRNAs. **(C)** Clustering heat map of differential circRNA expression. Red corresponds to upregulation; Green corresponds to downregulation. **(D)** Trend analysis of differential circRNAs. 3M, 1Y, and 3Y refer respectively to 3-month-old, 1-year-old, and 3-year-old.

Overall, 3982 (2,079 up-regulated; 1,903 down-regulated), 414 (201 up-regulated; 213 down-regulated), and 4,060 DE circRNAs (2,107 up-regulated; 1,953 down-regulated) were found in 3M vs 1Y, 1Y vs 3Y, and 3M vs 3Y groups, respectively (Figures 2A,C). Details about these circRNAs are available in Supplementary Tables 4–6. Among these, 44 circRNAs were co-expressed in testes from three age groups (Figure 2B). To characterize the dynamics of circRNA expression in developmental testes, we performed trend analysis of differentially expressed circRNAs in testes at three developmental stages. Here, we identified seven main circRNA profiles, where each represented a characteristic expression pattern (Figure 2D). Of these, three patterns, including profile 0 (continuously down-regulated), profile 1(firstly down-regulated followed by no change), and profile 6 (firstly up-regulated followed by no change) were significant.

### Validation of CircRNA Expression by qPCR

**FIGURE 3:**
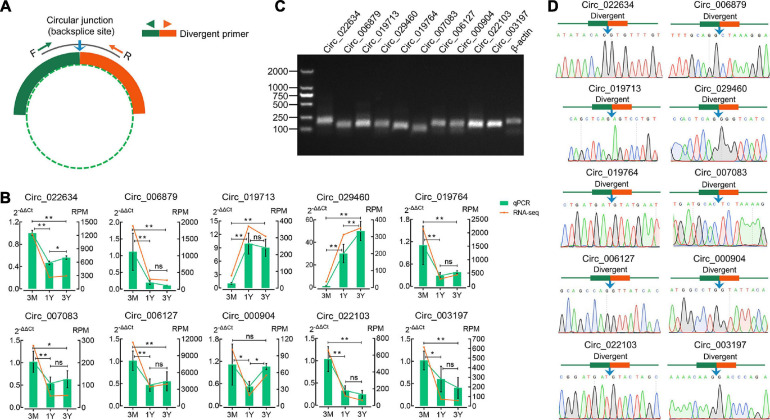
Verification of eight randomly selected circRNAs from RNA-Seq. **(A)** Divergent primers were designed for circRNA validation. **(B)** Comparison between qPCR and RNA-seq results. **(C)** Visualization of the PCR products on a 1.5% agarose electrophoresis gel. **(D)** Circular junctions were confirmed by Sanger sequencing. 3M, 1Y, and 3Y refer respectively to 3-month-old, 1-year-old, and 3-year-old. Two asterisks (**), one asterisk (*), and ns (non-significant) denote *P* < 0.01, *P* < 0.05, and *P* > 0.05, respectively.

The divergent primers for their junction sites from ten randomly selected DE circRNAs were used to determine the presence of these circRNAs (Figure 3A). The qPCR results suggested that expression profiles for these ten circRNAs were congruent with the RNA-sequencing results (Figure 3B). The 1.5% agarose gel electrophoresis shows the expected size of a single band for each selected circRNA (Figure 3C). The produced fragment was then sequenced by Sanger sequencing, and results showed the sequence information of circular junction for these circRNAs, obtained by Sanger sequencing, were exactly the same as circRNA sequencing (Figure 3D). These suggest that the sequencing data and expression of circRNAs identified in this study are reliable.

### Enrichment and Functional Annotation Analysis for Source Genes of DE circRNAs

**FIGURE 4:**
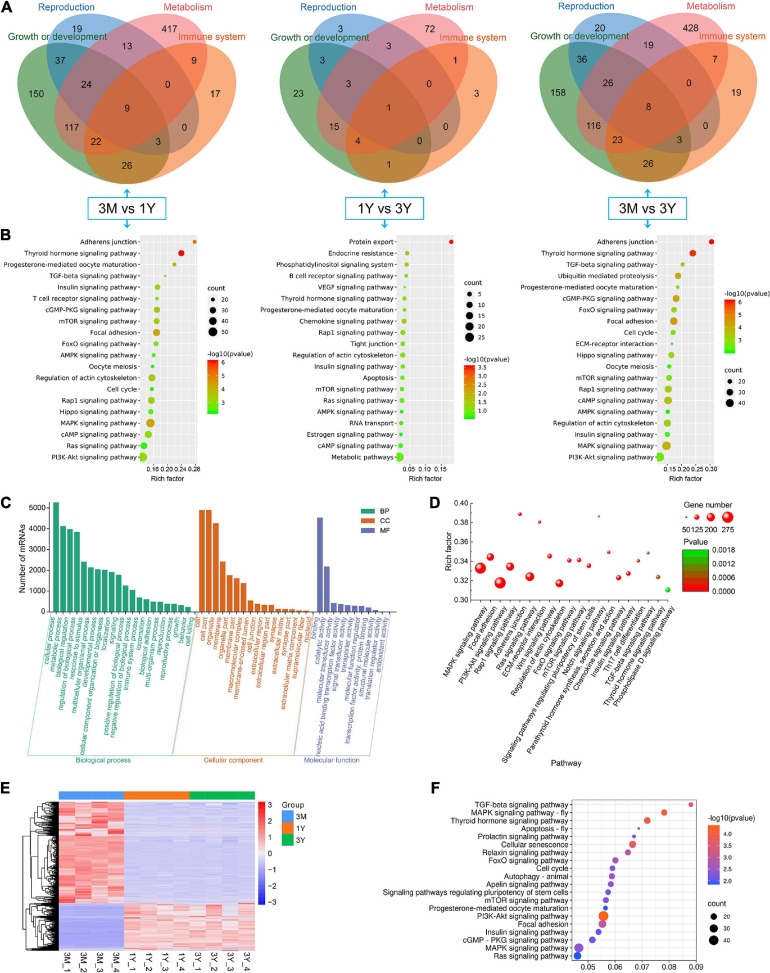
Functional annotation and enrichment analysis. **(A)** Functional classifcation and **(B)** KEGG pathway assessment of the source genes of differential circRNAs. **(C)** GO annotation and **(D)** KEGG enrichment of mRNAs in circRNA–miRNA–mRNA regulatory network. **(E)** Expression heatmap and **(F)** significantly enriched KEGG pathways of genes shared between DE circRNA source genes and DE mRNAs. 3M, 1Y, and 3Y refer respectively to 3-month-old, 1-year-old, and 3-year-old.

In 3M vs 1Y, 3,694 of the identified 3,982 DE circRNAs were derived from 2,079 source genes, and residual 288 circRNAs were derived from intergenic regions, having no source genes; in 1Y vs 3Y group, 370 of the identified 414 DE circRNAs were derived from 339 source genes, and residual 44 circRNAs were derived from intergenic regions, having no source genes; in 3M vs 3Y, 3,767 of the uncovered4060 DE circRNAs were derived from 2,119 source genes, and residual 293 circRNAs were originated from intergenic regions, having no source genes. To evaluate the biological roles of circRNAs, we mapped all the source genes for these DE circRNAs into GO terms in the GO resource and signaling pathways in the KEGG database. In total, 187, 73, and 182 GO terms for 3M vs 1Y, and 1Y vs 3Y, and 3M vs 3Y, respectively, was significantly enriched (Supplementary Table 7). These GO terms were mostly involved in biological processes such as reproduction, growth or development, immune system, and metabolism (Figure 4A). KEGG analysis indicated that the source genes for DE circRNAs, in 3M vs 1Y and 3M vs 3Y, were significantly enriched in cell cycle, oocyte meiosis, adherens junction, TGF-beta signaling, mTOR signaling, Hippo signaling, and other signaling pathways (Figure 4B); the source genes for DE circRNAs, in 1Y vs 3Y, were significantly enriched in tight junction, mTOR signaling, regulation of actin cytoskeleton, VEGF signaling, estrogen signaling pathway and other pathways (Figure 4B).

### CircRNA–miRNA–mRNA Network Construction and Functional Enrichment of mRNAs in Network

**FIGURE 5:**
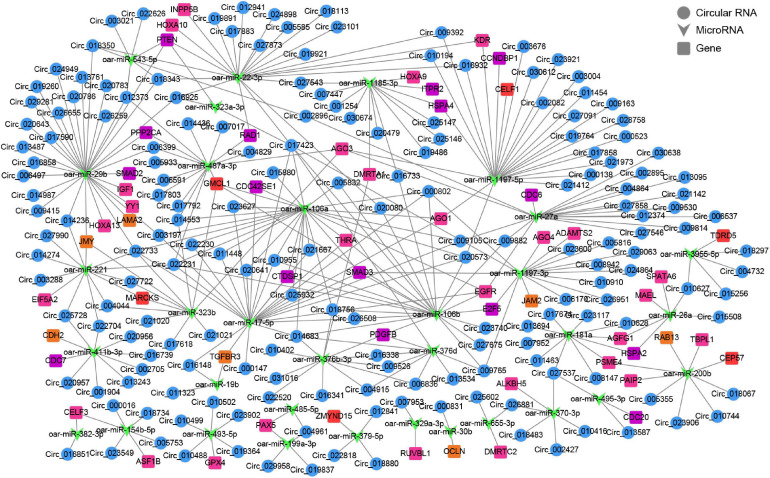
The circRNA–miRNA–mRNA interaction network. Blue circles represent circRNAs, green V shape represents miRNAs, and squares with different colors represent genes belonging to different functional classification (pink, spermatogenesis; orange, blood-testis barrier; red, germ cell development; purple, cell cycle or meiosis).

To further gain insights into the biological roles of these DE circRNAs, the ceRNA regulatory networks through the integration of our previous miRNA and mRNA sequencing data were constructed for circRNA–miRNA–mRNA interactions based on the “ceRNA hypothesis.” See Supplementary Table 8 for the list of mRNAs in the network. GO analysis showed that mRNAs derived genes in ceRNA network, on the biological processes, were mainly involved in, cellular component organization or biogenesis, reproduction, cellular process, development, as well as biological adhesion (Figure 4C); on the cellular components, were mainly included organelle, membrane, and extracellular matrix (Figure 4C); on the molecular function, mainly clustered with signal transducer activity, binding, catalytic activity, as well as transcription factor activity (Figure 4C). KEGG assessment indicated that these mRNAs derived genes were engaged in adherens junction, focal adhesion, ECM–receptor interaction, TGF-beta signaling, modulation of actin cytoskeleton, signaling cascades modulating pluripotency of stem cells, MAPK signaling, Wnt signaling, and so on (Figure 4D). The heatmap revealed that there were 1,335 genes which are shared by DE circRNA source genes and DE mRNAs (Figure 4E). Further functional analysis using GO and KEGG databases revealed these shared genes were primarily implicated in the biological processes such as reproduction, cellular process, development, growth and immunity (Supplementary Figure 1), and were significantly enriched in cell cycle, ECM–receptor interaction, TGF-beta signaling, mTOR, and other signaling pathway (Figure 4F and Supplementary Table 9), which were similar to the functional enrichment analysis results above. In the circRNA–miRNA–mRNA regulatory network, 28 known spermatogenesis-related genes (e.g., IGF1, DMRTC2, etc.) were potentially regulated by 143 circRNA–miRNA pairs covering 107 DE circRNAs and 24 DE miRNAs (Figure 5); 6 genes related to germ cell development were potentially regulated by 34 circRNA–miRNA pairs covering 33 DE circRNAs and 10 DE miRNAs (Figure 5); 16 cell cycle- or meiosis-related genes were potentially regulated by 140 circRNA-miRNA pairs covering 96 DE circRNAs and 18 DE miRNAs (Figure 5); 7 genes associated with blood–testis barrier (BTB) were potentially regulated by 45 circRNA–miRNA pairs covering 39 DE circRNAs and 9 DE miRNAs (Figure 5).

### Validation of Targeting Relationships Between circ_024949/circ_026259/IGF1 and oar-miR-29b and Their Tissue Expression Characteristics

**FIGURE 6:**
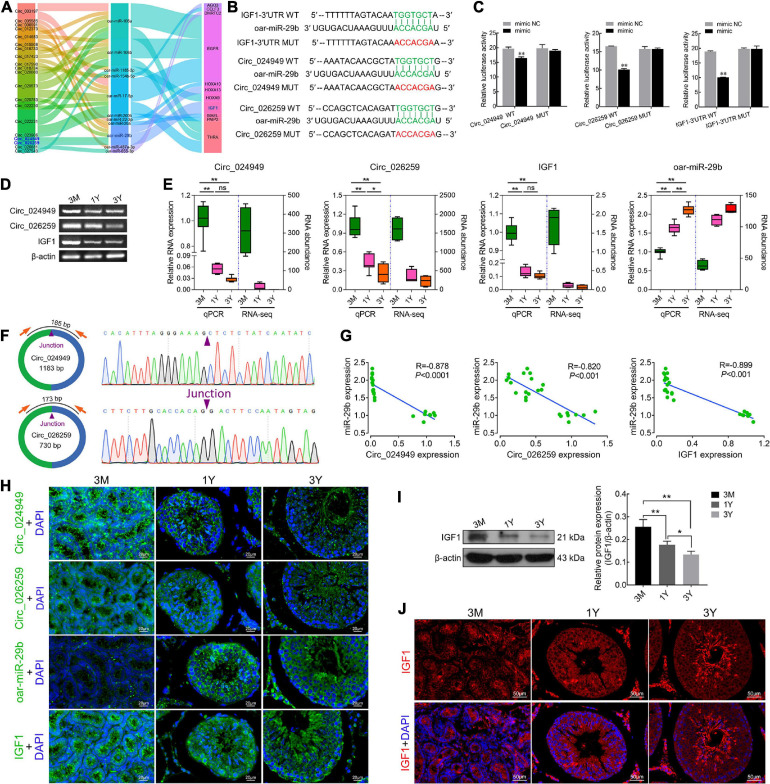
The targeting relationships between circ_024949/circ_026259/IGF1 and miR-29b as well as their expression correlations. **(A)** Sankey plot of the circRNA-miRNA-mRNA network enriched for spermatogenesis-related genes. **(B)** Putative binding sites revealed by bioinformatics analysis. **(C)** Dual-luciferase assays were used to validate the binding sites between circ_024949/circ_026259/IGF1-3′UTR and oar-miR-29b. **(D)** RT-PCR and **(E)** qPCR analysis for the relative RNA expression. **(F)** Divergent primers **(left panels)** and Sanger sequencing **(right panels)** used for the amplification of circular junctions of circ_024949 and circ_026259. **(G)** Correlation analysis between oar-miR-29b and circ_024949/circ_026259/IGF1 expression. **(H)** RNA fluorescence *in situ* hybridization analysis. **(I)** IGF1 protein expression assessed by Western blot. **(J)** Immunofluorescence staining for IGF1 protein. 3M, 1Y, and 3Y refer respectively to 3-month-old, 1-year-old and 3-year-old. Two asterisks (**), one asterisk (*), and ns (non-significant) denote *P* < 0.01, *P* < 0.05, and *P* > 0.05, respectively.

Based on the same expression trends between circRNAs and mRNAs and the inverse expression trends between circRNAs/mRNAs and miRNAs in testes at three development stages, circRNA–miRNA–mRNA regulatory network involved in spermatogenesis were further screened, which consists of 20 differentially expressed circRNAs, 11 miRNAs, and 11 mRNAs (Figure 6A). IGF1, a known important regulator of spermatogenesis (Potter and DeFalco, 2017; Neirijnck et al., 2019), is found to be present in this regulatory network, and potentially regulated by circ_026259/circ_024949/circ_012373-oar-miR-29b axis where circ_026259 and circ_024949 exhibit a higher abundance (Supplementary Tables 4–6) and a more significant relationship (circ_026259/IGF1, *P* = 0.0078; circ_024949/IGF1, *P* = 0.0169) compared to circ_012373 (*P* = 0.0303). Based on these considerations, we selected circ_026259 and circ_024949 to verify their targeting relationships between circ_024949 and oar-miR-29b, circ_026259 and oar-miR-29b, and IGF1 and oar-miR-29b, and to assess their expression patterns.

The putative binding sites of circ_024949, circ_026259 and IGF1-3′UTR with oar-miR-29b are provided in Figure 6B. Dual-luciferase reporter assay indicated that oar-miR-29b significantly suppressed the luciferase activities for co-transfecting with wild types of circ_024949, circ_026259, or IGF1-3′UTR compared with oar-miR-29b negative control (*P* < 0.01), while no effect on the mutant types of circ_024949, circ_026259 or IGF1-3′UTR (*P* < 0.05) (Figure 6C). These results initially confirmed the direct interactions between circ_026259/circ_024949/IGF1 and oar-miR-29b. Subsequently, RT-PCR and qPCR results demonstrated that the relative RNA expression of circ_024949, circ_026259, and IGF1 gradually decreased (Figures 6D,E), while that of oar-miR-29b progressively increased with the advance of ages, showing excellent agreement with our results obtained by RNA-seq (Figure 6E). Sanger sequencing corroborated the back-splicing junction sites in the qPCR products of circ_024949 and circ_026259 amplified by divergent primers (Figure 6F). Pearson’s correlation analysis indicated that there was a strong negative correlation between circ_024949 and oar-miR-29b, circ_026259 and oar-miR-29b, oar-miR-29b and IGF1 (R = −0.878, −0.820, −0.899, *P* < 0.001) (Figure 6G). *In situ* hybridization revealed that RNA distribution patterns for circ_024949, circ_026259, oar-miR-29b and IGF1 were very similar in developmental testes and extensively existed in various testicular cells (such as germ cells, Leydig cells, and Sertoli cells) (Figure 6H). Consistent with the mRNA level, IGF1 protein expression gradually decreased with age (Figure 6I), and localized ubiquitously to various germ cells as well as Leydig cells and Sertoli cells and, with intense expression in spermatozoa and the cytoplasm of Leydig cells from 1Y and 3Y, as well as the seminiferous epithelium from 3M (Figure 6J).

### Circ_026259 Regulated IGF1 Expression by Targeting oar-miR-29b in Testicular Sertoli Cells

**FIGURE 7:**
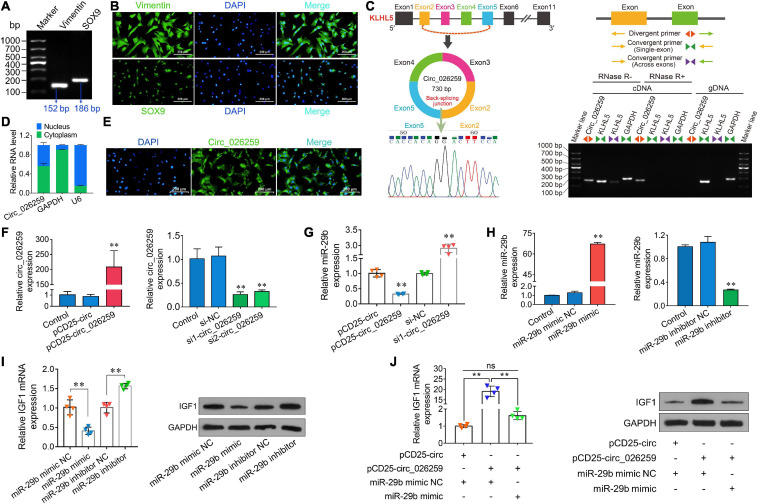
Characterization and expression analysis of circ_026259 and effects of circ_026259 on oar-miR-29b and IGF1 expression in sheep Sertoli cells. **(A)** RT-PCR and **(B)** immunofluorescence assays of marker genes (Vimentin and SOX9) were performed to identify the primary Tibetan sheep Sertoli cells. **(C)** The structure characteristics of circ_026259. **(Left upper panel)** Schematic illustration showing circularization of circ_026259 originated from exon 2 to exon 5 of the ovine KLHL5 gene. **(Right panel)** Divergent primers amplified circ_026259 in cDNA in Sertoli cells with or without RNase R, but not genomic DNA (gDNA). **(Left lower panel)** Sanger sequencing confirmed the back-splicing junction of circ_026259. **(D)** qPCR detection and **(E)** FISH localization of circ_026259 in Sertoli cells. **(F)** Examination of circ_026259 overexpression **(left panel)** and knockdown **(right panel)** efficiency by qPCR. **(G)** Effects of overexpression and knockdown of circ_026259 on expression of oar-miR-29b. **(H)** Examination of oar-miR-29b overexpression **(left panel)** and knockdown **(right panel)** efficiency by qPCR. **(I)** Effects of overexpression and knockdown of oar-miR-29b on IGF1 mRNA and protein expression. **(Left panel)** IGF1 mRNA levels relative to GAPDH. **(Right panel)** the representative images showing IGF1 protein expression levels obtained using Western blot. **(J)** Circ_026259 overexpression can rescue the suppressive effects of oar-miR-29b on IGF1 mRNA and protein expression in Sertoli cells. **(Left panel)** the relative mRNA levels of IGF1. **(Right panel)** the representative Western blot images showing IGF1 protein expression. ^∗∗^ denotes *P* < 0.01.

On the basis of our above experiment results and prior literature documenting a crucial role for IGF1 in Sertoli cells which modulates cell proliferation (Cannarella et al., 2018; Johnson et al., 2020), we thus chose to focus on Sertoli cells for more detailed investigation in ovine testicular cells. Here we isolated ovine primary Sertoli cells using two-step enzymatic digestion and the isolated primary cells strongly expressed the Sertoli cell-specific markers Vimentin and SOX9 (Figures 7A,B), suggesting successful isolation of Sertoli cells from sheep testis tissues. Circ_026259 was therefore chosen for subsequent oar-miR-29b/IGF1 axis analysis in Sertoli cells since circ_026259 showed a higher RNA abundance compared to circ_024949, either as RNA-seq and qPCR data from testicular tissues or qPCR data from Sertoli cells (data not shown). Bioinformatics revealed that circ_026259, with 730 bp in length, is derived from the exon 2, exon 3, exon 4, and exon 5 of KLHL5 gene, and the end of exon 2 and exon 5 was back-spliced to form the circular structure (Figure 7C). We further confirmed its circular character using the RNase R digestion method, RT-PCR and Sanger sequencing (Figure 7C), corroborating the existence of circ_026259 in Sertoli cells. Circ_026259 was mainly located in the cytoplasm of Sertoli cells but expression was also found in the nucleus (Figures 7D,E), based on nuclear and cytoplasmic fractionation and FISH, which suggest that circ_026259 may exert its action through ceRNA regulation pattern.

To explore whether circ_026259 regulates the expression of IGF1 in Sertoli cells, circ_026259 overexpression and knockdown experiments were carried out *in vitro*. The transfection of PCD25-circ_026259 plasmid significantly enhanced the expression of circ_026259 in Sertoli cells (Figure 7F). We used two siRNAs targeting the junction site of circ_026259 and showed that circ_026259 expression was effectively decreased by transfection with two siRNAs (si1-circ_026259, si2-circ_026259) in comparison with the control and si-NC groups (Figure 7F), where si1-circ_026259 exhibited more potent knockdown effects and was therefore selected for subsequent investigation. We next examined the expression of oar-miR-29b upon overexpression or knockdown of circ_026259 in Sertoli cells, and the results demonstrated that the expression of oar-miR-29b was significantly inhibited by overexpressing circ_026259, which was significantly enhanced through circ_026259 knockdown (Figure 7G). Overexpression of oar-miR-29b significantly suppressed the expression of IGF1, whereas its knockdown significantly increased IGF1 expression (Figures 7H,I). Additionally, circ_026259 overexpression can restore the repression of IGF1 expression at mRNA and protein levels by oar-miR-29b to certain extent (Figure 7J). Together, this series of experiments support that circ_026259 as a miR-29b sponge to indirectly modulate the expression of IGF1.

## Discussion

The testis is a strongly transcriptionally active organ whose main functions is to produce sperm through spermatogenesis (Witt et al., 2019). CircRNAs, as an abundant non-coding RNA in the eukaryotic transcriptome, are important modulators involved in modulating expression of genes at the transcriptional, post-transcriptional as well as translational levels and miRNA functions (Ebbesen et al., 2017). Herein, we first identified and characterized the expression patterns of circRNAs in developmental sheep testes via high-throughput sequencing, as well as bioinformatics analysis. Here, 3,982, 414, and 4,060 DE circRNAs were identified in 3M vs 1Y, 1Y vs 3Y, and 3M vs 3Y groups, respectively. These differential circRNAs were clustered into seven overall expression patterns of which three patterns involved 3,485 DE circRNAs showed significant clustering. Among these, with the increase of age during testicular development, 190, 1,271, and 2,024 circRNAs exhibited the expression trends of continuous decreasing, of first decreasing followed by no change, and of increasing followed by no change, respectively. The qPCR analysis and Sanger sequencing were done to validate circRNA sequencing data, and results support that circRNAs obtained in this study are of high quality and reliable.

To explore the roles of circRNAs in developmental sheep testis, functional analysis of the source genes of DE circRNAs was conducted. GO functional annotation indicated that circRNA source genes mainly participated in biological processes including reproduction, growth and development. For instance, in 3M vs 1Y and 3M vs 3Y, 8 common DE circRNAs were generated from DAZL and BOLL that belong to deleted in azoospermia (DAZ) gene family associated with germ cell development and spermatogenesis (Fu et al., 2015); 29 DE circRNAs were derived from 15 genes coding for cell division cycle-related proteins (e.g., CDC14B, CDC20B, CDC6, GAK, CDK6, CDK12-13, CDK17, etc.). In 1Y vs 3Y, differential circRNA circ_025151 was generated from SMAD5, a gene has been demonstrated to promote cell proliferation and inhibit apoptosis of Sertoli cells in pigs (Luo et al., 2020); circ_012965 was derived from SPIN1 that is a gene associated with meiotic progression and meiotic cell cycle in mammals (Choi et al., 2019). It is suggestive of a function for these circRNAs in testicular cell cycle progression and cell development thereby participating in the regulation of spermatogenesis and testis development.

Notably, in this study, some of circRNA source genes were linked to the annotation correlated with the function of the immune system. Besides spermatogenesis and steroidogenesis, the testis has also been considered as an immunological privileged organ where immune responses are limited to protect immunogenic germ cells from an immune attack and to sustain the immune homeostasis required for normal spermatogenesis (Zhao et al., 2014). The blood-testis barrier has long been known to be a crucial part of the testicular immune privilege to sequester auto-antigenic germ cells and is one of the tightest tissue barrier present in mammals, which is primarily formed by the integrins (Yan et al., 2007) and junctional complex (Smith and Braun, 2012; Mruk and Cheng, 2015) between Sertoli cell-Sertoli cell, Sertoli cell-matrix and Sertoli cell-germ cell (e.g., tight junctions, adherens junctions) to sustain the barrier integrity and function. As expected, we found that 12 DE circRNAs shared by 3M vs 1Y and 3M vs 3Y groups were derived from eight genes (CTNNA1, NECTIN3, GJC1, TNS1, STRN, SMAD2, NUMBL, and TGFB2) associated with cell junctions, including cell-cell junction, cell-substrate junction and adherens junction. Twelve common DE circRNAs were derived from genes encoding integrin molecules, including ITGA4, ITGA9, ITGAL, ITGAV, ITGB1, ITGB8, ITGB3BP, and ITGBL1.

Moreover, the intratesticular interstitial space interspersed between the seminiferous tubules contains a variety of immune cell population of immune system engaged in the regulation of immune responses against microbial infections: macrophages, lymphocytes (mainly T cells), dendritic cells, as well as mast cells (Pérez et al., 2013). During testicular development, these immune cells contribute to sustaining immune homeostasis via the involvement of immune tolerance, local immunosuppression and decreased immune activation (Meinhardt and Hedger, 2011). In this study, in 3M vs 1Y and 3M vs 3Y, 77 shared source genes for DE circRNAs were linked with immune response based on GO annotation. For example, circ_001819 and circ_001824 derived from CBLB gene and circ_023878 and circ_023884 derived from GLI3 gene were annotated to negative regulation of lymphocyte activation; six DE circRNAs (circ_025570, circ_021583, circ_004726, circ_028039, circ_028043 and circ_028044) generated from four genes: MEF2C, BCL2L11, STAT5B and HIF1A, were annotated as participating in lymphocyte/leukocyte homeostasis. Compared with 3M vs 1Y and 3M vs 3Y, however, there were only 10 source genes for DE circRNAs, in 1Y vs 3Y, were found to be implicaed in immune response based on GO annotation, which might be responsible for no significant changes in intratesticular immune microenvironment in post-pubertal (sexually mature and adult) sheep testis. One of the major functions of testicular immune privilege is to protect germ cells (especially meiotic germ cell including the spermatozoa) from systemic auto-immune attack (Meng et al., 2011). As we all know, prepubertal testis do not undergo spermatogenesis until pubertal onset when wave of spermatogenesis begins, including mitotic proliferation of spermatogonia and initiation of the meiotic program of spermatocytes, to allow testis to produce mature spermatozoa up to old age.

Likewise, a KEGG analysis domonstrated that the source genes for DE circRNAs, in 3M vs 1Y and 3M vs 3Y, were mostly enriched in the signaling pathways associated with reproduction [e.g., oocyte meiosis, insulin signaling, VEGF signaling (Kang et al., 2015), progesterone-mediated oocyte maturation, etc.], spermatogenesis [e.g., mTOR signaling (Jesus et al., 2017), Hippo signaling (Zhang G.M. et al., 2019), TGF-beta signaling (Fan et al., 2012), cell cycle, etc.], BTB [e.g., adherens junction (Mruk and Cheng, 2015; Stanton, 2016), modulation of actin cytoskeleton (Mruk and Cheng, 2015), focal adhesion (Mruk and Cheng, 2015), ECM-receptor interaction (Siu and Cheng, 2008), etc.], and immune responses [e.g., T cell receptor signaling, PI3K-Akt signaling, leukocyte transendothelial migration, etc.]; in 1Y vs 3Y, were mainly enriched in the signaling pathways associated with reproduction [e.g., estrogen signaling, progesterone-mediated oocyte maturation, VEGF signaling (Kang et al., 2015), etc.], BTB [e.g., tight junction (Mruk and Cheng, 2015; Stanton, 2016), modulation of actin cytoskeleton (Mruk and Cheng, 2015), etc.], and immune responses (e.g., chemokine signaling, B cell receptor signaling, etc.].

Multiple lines of evidence demonstrated that one of the primary functions of circRNAs is to act as miRNA sponges to indirectly regulate expression of downstream target gene for miRNAs (Zhong et al., 2018). Thus, we combined with previous miRNA-seq and mRNA-seq data and further analyzed circRNA–miRNA–mRNA interaction network based on ceRNAs to reveal the functions involved by these DE circRNAs. The results revealed by GO analysis indicated that these target genes were assigned to several main biological processes, including cellular process, development, biological adhesion, and reproduction. By analyzing the results of KEGG pathway enrichment, we also discovered that they significantly enriched in pathways linked with spermatogenesis or testicular immune privilege, such as Wnt signaling (Kang et al., 2015), TGF-beta signaling (Fan et al., 2012), signaling cascades modulating pluripotency of stem cells, modulation of actin cytoskeleton (Mruk and Cheng, 2015), adherens junction (Mruk and Cheng, 2015; Stanton, 2016), focal adhesion (Mruk and Cheng, 2015), ECM–receptor interaction (Siu and Cheng, 2008), and chemokine signaling. Additionally, similar functional enrichment results were found in GO analysis and KEGG analysis of the common genes between differentially expressed circRNAs’ souce genes and differentially expressed mRNAs.

According to GO and KEGG analysis of mRNAs in ceRNA network, the target genes related to spermatogenesis, blood-testis barrier, germ cell development, cell cycle or meiosis were selected to build a circRNA–miRNA–mRNA network. As a result, there were 259 circRNA–miRNA pairs and 97 miRNA–mRNA pairs in the constructed network, involving 180 circRNAs, 33 miRNAs, and 57 genes. Among these, IGF1, a known testicular development gene (Cannarella et al., 2018; Neirijnck et al., 2019), was predicted to be modulated by circ_024949/circ_026259-oar-miR-29b signals, and the subsequent dual-luciferase assays confirmed that circ_024949/circ_026259/IGF1 shared the same oar-miR-29b target site. It has been reported that IGF1 is widely expressed in various cell types in the testis, containing germ cells, Leydig cells, and Sertoli cells to modulate their development and functions (Potter and DeFalco, 2017; Cannarella et al., 2018). Similarly, our results indicated that at the protein level, IGF1 protein was wildly existed in various germ cells, along with existence in Leydig cells and Sertoli cells, suggestive of its multiple roles in development and maturation of germ cells as well as functional maintenance of testicular somatic cells. miR-29b, a member of miR-29 family, has been documented to be closely related to male fertility (Menezes et al., 2020). This study showed that oar-miR-29b was remarkably up-regulated in post-pubescent sheep testis (1- and 3-year-old) relative to pre-pubescent (3-month-old), which is in agreement with previous reports in canine testis (Kasimanickam and Kasimanickam, 2015). Consistent with the results indicated in the RNA-seq data, however, RT-PCR and qPCR results revealed that the exepression of circ_024949, circ_026259 and IGF1 decreased gradually with development from 3-month-old to 3-year-old testes, suggesting that their expression negatively correlated with oar-miR-29b. The subsequent *in situ* hybridization analysis revealed that circ_024949, circ_026259, oar-miR-29b and IGF1 were all located in various germ cells, Leydig cells and Sertoli cells, which was largely consistent with a previous report on the subcellular localization patterns of testicular IGF1 mRNA (Yuan et al., 2018). This further implied the interactions between either circ_024949, circ_026259, or IGF1 and oar-miR-29b, as well as their potential modulation for the development of testicular germ and somatic cells.

Given the existing literature outlining the importance of IGF1 in Sertoli cell development (Cannarella et al., 2018; Johnson et al., 2020) and the above findings, we successfully isolated ovine primary Sertoli cells to further evaluate the regulatory effect of circ_026259 (having a higher expression compared to circ04949 in Sertoli cells) on IGF1 expression through oar-miR-29b in testicular cells. The results from the study by overexpression or knockdown of circ_026259 and oar-miR-29b demonstrated the positive effects of circ_026259 on IGF1 mRNA and protein expression via repressing oar-miR-29b in Sertoli cells.

Additionally, IGF1, as a growth factor secreted by testicular cells (especially Leydig cells), may possess immunosuppressive property in mammalian testis (Pérez et al., 2013; Potter and DeFalco, 2017). A recent study documented that IGF1 functions in the intestinal immune homeostasis (Zheng et al., 2018). These reports together raise the possibility that IGF1 expression may also contribute to the maintenance of immunologic homeostasis during testis development, but whether IGF1 is functional in this and the mechanistic details of regulation by which IGF1 remain to be elucidated. Regardless, these data somewhat enrich our understanding of the functionality of IGF1 in sheep testis and how its expression is regulated.

In conclusion, this is the first documentation elucidating circRNA dynamic expression patterns across three reproductive stages in postnatal sheep testes and describing circRNA-associated miRNA-gene network implicated in testicular function; as well as the first to confirm that circ_024949 and circ_026259 were likely to jointly regulate IGF1 expression through direct targeting of oar-miR-29b, and therefore had multiple functions in development of germ cells and functional maintenance of Sertoli cells and Leydig cells during ram spermatogenesis. Further, circ_026259, a novel circular RNA derived from KLHL5 exons, was confirmed to negatively modulate IGF1 expression by inhibiting oar-miR-29b in sheep Sertoli cells. These findings provide novel vision and direction for the future exploration of the regulatory mechanisms of testicular development and function in sheep.

## Data Availability Statement

RNA-seq data has been deposited to the SRA database in NCBI (accession numbers: SRR11348536-SRR11348547).

## Ethics Statement

The animal study was reviewed and approved by Laboratory Animal Welfare and Ethics Committee of Gansu Agricultural University. Written informed consent was obtained from the owners for the participation of their animals in this study.

## Author Contributions

TL and YM conceived and designed the study. XW, HW, and HJ collected the samples. TL, RL, XW, and HW performed the experiments and analyzed the data. TL wrote the manuscript. XZ, YG, and YM contributed to the revisions of the manuscript. All authors read and approved the manuscript.

## Conflict of Interest

The authors declare that the research was conducted in the absence of any commercial or financial relationships that could be construed as a potential conflict of interest.

## Footnotes

1 http://www.rproject.org/

2 https://sourceforge.net/projects/mireap/

3 http://www.targetscan.org

4 http://miranda.org.uk/

## Supplementary Material

Supplementary Figure 1 GO annotation of the genes shared between DE circRNA source genes and DE mRNAs.

Supplementary Table 1 RNA sequences used for transfection.

Supplementary Table 2 A list of primers used in PCR analysis.

Supplementary Table 3 Summary statistics of circular RNA sequencing data.

Supplementary Table 4 List of differentially expressed circRNAs between 3M and 1Y.

Supplementary Table 5 List of differentially expressed circRNAs between 3M and 3Y.

Supplementary Table 6 List of differentially expressed circRNAs between 1Y and 3Y.

Supplementary Table 7 GO annotation of source genes for differentially expressed circRNAs.

Supplementary Table 8 The mRNA expression list in ceRNA network.

Supplementary Table 9 KEGG analysis of the genes shared between DE circRNA source genes and DE mRNAs.
