# Supplementary figures and images for: Unraveling Stage-Dependent Expression Patterns of Circular RNAs and Their Related ceRNA Modulation in Ovine Postnatal Testis Development
Source: Front Cell Dev Biol. 2021 Mar 19;9:627439. doi: 10.3389/fcell.2021.627439 (PMC8017185; doi:10.3389/fcell.2021.627439)

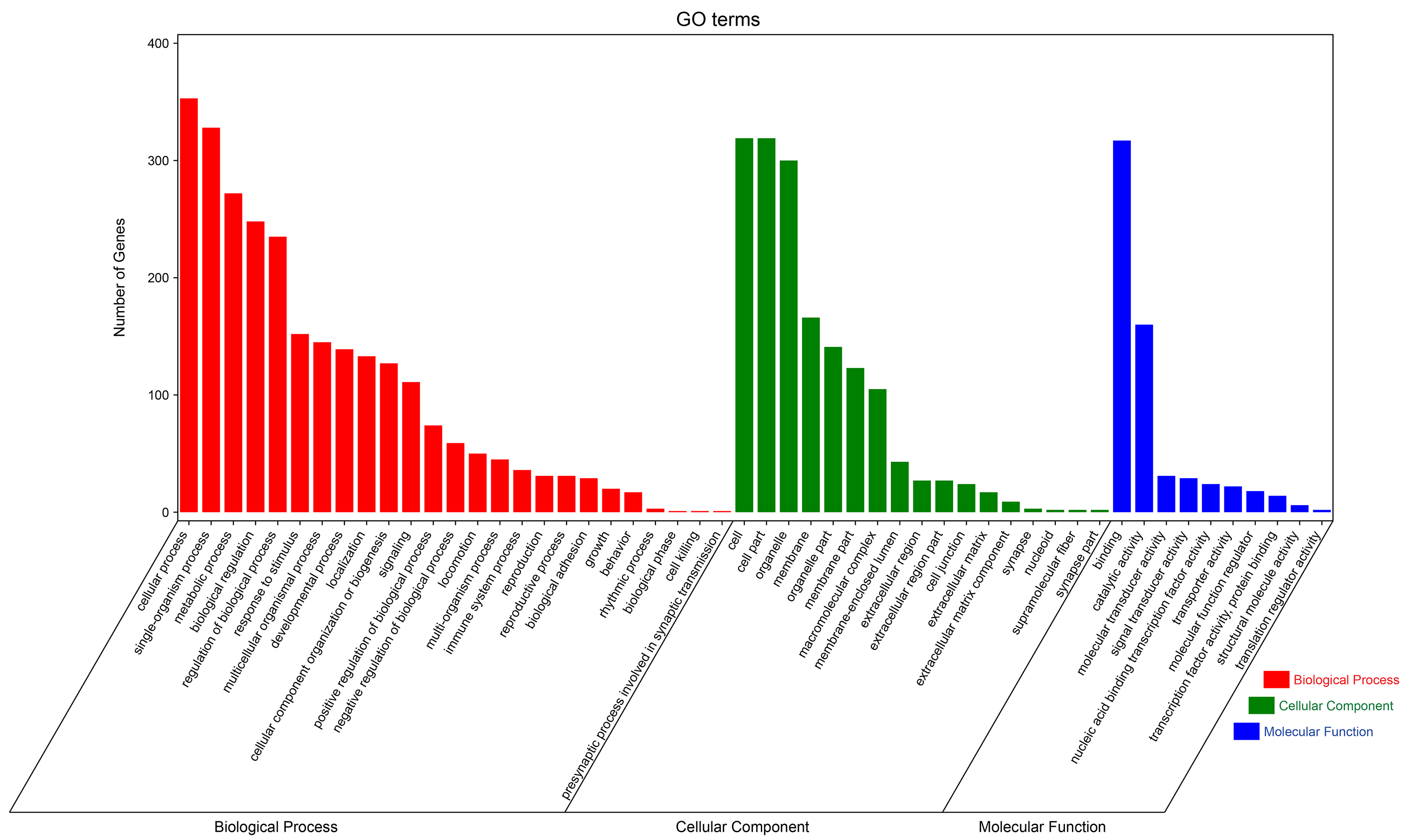

Supplement: Supplementary Figure 1 — GO annotation of the genes shared between DE circRNA source genes and DE mRNAs. [file Image_1.TIF]
